# Correction of breast deformities after breast-conserving surgery in Chinese women with breast cancer: a narrative review

**DOI:** 10.3389/fonc.2025.1648679

**Published:** 2025-09-18

**Authors:** Zejun Wu, Long Zhong, Jiaxu Gu, Hongqiang Xie, Fan Guo

**Affiliations:** ^1^ Department of Breast Surgery, Third Hospital of Shanxi Medical University, Shanxi Bethune Hospital, Shanxi Academy of Medical Sciences, Tongji Shanxi Hospital, Taiyuan, China; ^2^ Shenzhen University Medical School, Shenzhen University, Shenzhen, China; ^3^ Department of Urology, Peking university Shenzhen hospital, Shenzhen, China; ^4^ Institute of Urology, Shenzhen Peking University-The Hong Kong University of Science and Technology Medical Center, Shenzhen, China; ^5^ Shenzhen Clinical Research Center for Urology and Nephrology, Peking University Shenzhen Hospital, Shenzhen, China; ^6^ Peking university, Beijing, China; ^7^ Department of Dermatology, Peking university Shenzhen hospital, Shenzhen, China; ^8^ Department of Intensive Care Unit, Nanjing Drum Tower Hospital Clinical College of Xuzhou Medical University, Nanjing, China

**Keywords:** breast-conserving surgery, postoperative deformity, willingness for correction, influencing factors, breast cancer

## Abstract

Breast-conserving therapy (BCT) is a key treatment option for early-stage breast cancer, balancing oncologic control with cosmetic outcomes. However, postoperative breast deformities occur in approximately 25% to 30% of cases, significantly impacting patients’ body image and psychological well-being. Although advances in oncoplastic surgery have demonstrated the safety and efficacy of corrective procedures—effectively improving breast appearance, alleviating anxiety, and enhancing quality of life—the acceptance rate of such surgeries remains relatively low in China. This reluctance is influenced by multiple factors, including personal characteristics, social support, disease-related features, and treatment experiences. This article reviews the necessity and safety of deformity correction after BCT, as well as the factors affecting patients’ willingness to undergo such procedures. The aim is to help clinicians identify suitable candidates for corrective surgery, promote multidisciplinary interventions and decision-making support, and ultimately improve the correction rate and overall quality of life for breast cancer patients.

## 1 Background

According to the latest data from the International Agency for Research on Cancer (IARC) released in 2020, breast cancer has surpassed lung cancer as the most commonly diagnosed malignancy among women worldwide, accounting for an estimated 11.7% of new cancer cases (1). Cancer statistics in China similarly indicate that breast cancer ranks first among newly diagnosed malignancies in women and continues to show an upward trend in incidence (2). Currently, surgery remains the primary treatment modality for early-stage breast cancer, with the most common options being mastectomy and breast-conserving therapy (BCT) (3). Multiple large-scale clinical trials have confirmed that patients undergoing BCT combined with radiotherapy achieve long-term survival rates comparable to those who receive total mastectomy (4). BCT not only ensures oncologic safety but also preserves breast contour, offering cosmetic advantages that significantly enhance patients’ quality of life and psychological well-being. As a result, BCT has gradually become the mainstream surgical choice for early-stage breast cancer.

However, recent studies have reported that approximately 25% to 30% of patients develop varying degrees of breast deformities after undergoing conventional breast-conserving surgery (CBCS). The more pronounced the deformity, the higher the risk of depressive symptoms—reported at 16.2% for mild asymmetry, 18.0% for moderate, and up to 33.7% for severe asymmetry (5). Such deformities not only compromise physical appearance but also substantially impair quality of life. Corrective procedures can restore breast aesthetics and improve overall well-being. Nevertheless, the rate of corrective surgery among Chinese women remains relatively low. In light of this, the present article aims to provide a comprehensive review of the safety, necessity, and influencing factors associated with post-BCT breast deformity correction, with the goal of informing clinical decision-making.

## 2 Material and methods

We conducted a literature search in PubMed from inception to February 2025. The search strategy included a combination of keywords and medical subject headings (MeSH) related to “oncoplastic breast-conserving reconstruction,” “post–breast-conserving surgery,” “deformity correction,” “perforator flap,” “willingness for correction,” and “influencing factors.” Articles were screened for relevance based on titles and abstracts, and full texts were reviewed when necessary. Additional references were identified through manual searching of the bibliographies of selected articles.

### 2.1 Current status and impact of breast deformities after breast-conserving surgery

In recent years, with improvements in early breast cancer screening and evolving treatment paradigms, breast-conserving surgery (BCS) has seen increasingly widespread use in clinical practice. However, the incidence of postoperative breast deformities remains high. These deformities—ranging from asymmetry and contour depressions to scarring—compromise the aesthetic integrity of the breast and may lead to psychological distress and social withdrawal (6), thereby significantly impairing patients’ quality of life. A systematic review by Berry et al. reported that the incidence of poor cosmetic outcomes following BCS ranges from 5% to 30% (7). Although the introduction of oncoplastic techniques has aimed to mitigate the disadvantages associated with conventional BCS (8), postoperative deformities still persist. Iwuchukwu and colleagues reviewed the literature and noted that 5% to 14% of patients continued to experience suboptimal cosmetic outcomes even after oncoplastic surgery (9). This suggests that while oncoplastic approaches may reduce the risk of aesthetic complications compared to traditional BCS, they do not eliminate them entirely (6). Moreover, adjuvant radiotherapy often induces tissue fibrosis and skin pigmentation, further contributing to the development of postoperative deformities (10). As survival rates improve, there is a growing clinical emphasis on both functional and aesthetic restoration. Accordingly, the prevention and correction of breast deformities after BCS have become key areas of focus in breast surgery.

### 2.2 Safety and surgical options for correcting breast deformities after breast-conserving surgery

Currently, a wide range of surgical techniques are available for correcting breast deformities following BCS. With advances in reconstructive surgery, local perforator flaps such as the anterior intercostal artery perforator (AICAP) flap, thoracodorsal artery perforator (TDAP) flap, and lateral intercostal artery perforator (LICAP) flap have proven effective in repairing post-BCS breast defects. Studies have shown that flap techniques can safely restore breast contour (11). Li Xie reported that among patients who underwent chest wall perforator flap reconstruction, only one case of tumor recurrence was observed during a median follow-up period of 14.5 months, with no reported deaths (12). Pujji reviewed 11 studies published between 1990 and 2020 involving a total of 432 cases using perforator flap techniques. After a mean follow-up period of 21 months (range 1 – 49 months), only one case of local recurrence was observed (13). Similarly, in a study by Roy, 105 patients underwent lateral chest wall perforator flap reconstruction, with a reported local recurrence rate of 2% and an overall survival rate of 94.8% during a median follow-up of 4.5 years (14).

Autologous fat grafting (AFG) is another well-established method for addressing localized volume deficits. Evidence suggests that AFG does not increase the risk of cancer recurrence (15). A comprehensive analysis of all oncoplastic breast reconstruction cases using AFG—identified through searches of PubMed, Embase.com, Wiley/Cochrane Library, and Web of Science from January 1996 to November 2014—found no evidence of cancer recurrence or significant complications, affirming the oncologic safety of fat grafting (16). Federico Lo Tort conducted a meta-analysis that included 40 studies, encompassing 7,619 patients who underwent autologous fat grafting (AFG), with a reported overall local recurrence rate (LRR) of 3.15%. In comparison, among 6,459 patients who did not receive AFG, the LRR was 5.3%. The analysis found no significant association between AFG and an increased risk of local recurrence, further confirming that autologous fat grafting is a safe and feasible adjunctive reconstructive technique (17).

For patients presenting with macromastia or breast asymmetry following BCS, reduction mammaplasty and mastopexy are often recommended. These procedures have not been shown to increase the risk of tumor recurrence, and their complications are generally controllable, supporting their safety (18). In a study investigating contralateral breast adjustment following breast-conserving surgery, 77 patients underwent immediate oncoplastic reconstruction along with contralateral symmetrization. During a 5-year follow-up period, the local recurrence rate was 4.1%. These findings suggest that contralateral symmetrization in the context of oncoplastic surgery is a safe and effective therapeutic strategy (19). In a study by Maurizio B. Nava and colleagues, patients were divided into three groups based on the type of contralateral breast reshaping technique: Group 1 underwent mastopexy or reduction mammaplasty with an inferior dermoglandular flap; Group 2 underwent mastopexy or reduction without the flap; and Group 3 did not receive any contralateral reshaping. The incidence of contralateral metachronous breast cancer was assessed, and no statistically significant difference was found among the groups (8.6% in Group 1, 8.6% in Group 2, and 5.7% in Group 3; *p*=0.87). These findings indicate that contralateral reshaping procedures such as mastopexy and reduction are highly safe following breast reconstruction. Additionally, parenchymal rearrangement during oncoplastic surgery does not impair the ability of imaging techniques to detect subsequent malignancies (20). Similarly, Muir’s study confirmed that breast changes following reduction mammaplasty do not significantly interfere with the detection of cancer on mammography (21). Collectively, these findings indicate that surgical correction of post-BCS deformities is both safe and clinically effective. Given the variations in postoperative complications among different surgical techniques, a summary is presented in **Table 1**.

**Table 1:** Common complications and reported incidence of deformity correction techniques after breast-conserving surgery.

Surgical technique	Main complications	Reported incidence (from included studies)	References
Autologous fat grafting (AFG)	Fat necrosis, oil cysts, volume resorption	No recurrence increase observed; Local recurrence rate: 3.15% (AFG group) vs 5.3% (non-AFG)	(16, 17)
Local perforator flap	Partial flap necrosis, wound dehiscence, seroma	Flap necrosis: rare In a study of 432 cases: 1 local recurrence during average 21-month follow-up	(13, 14)
Latissimus dorsi (LD) flap	Donor site morbidity (pain, seroma), scar	No specific complication rate reported; used for large defects	(14)
Reduction mammoplasty/Mastopexy	Nipple-areola sensory loss, asymmetry, wound healing delay	Local recurrence rate: 4.1% during 5-year follow-up (n=77)	(19)
Contralateral symmetry procedures	Extended surgery time, wound tension	No significant difference in contralateral cancer rate: 8.6% vs 5.7% (p=0.87)	(20)
Implant-based techniques (rare in BCT correction)	Capsular contracture, radiation-related complications	Higher complication rate post-radiotherapy: 68% vs. 31% (p=0.006)	(56)

### 2.3 Clinical necessity and outcome evaluation of deformity correction after breast-conserving surgery

With the ongoing refinement of breast-conserving techniques, the concept of oncoplastic surgery has emerged as a critical advancement in the field. According to the American Society of Breast Surgeons, oncoplastic techniques are generally categorized into two approaches: volume displacement and volume replacement (8). Volume displacement involves redistributing the remaining breast tissue to fill the defect after resection, while volume replacement entails augmenting the defect using flaps or implants to restore volume after partial mastectomy. The Clough classification further divides oncoplastic procedures into Level I and Level II. Level I procedures involve the resection of less than 20% of breast tissue and typically include minor reshaping techniques such as glandular mobilization and nipple–areola complex repositioning. Level II procedures address more extensive resections (20%–50% of breast tissue) and require volume displacement or replacement to restore breast contour (22). A Spanish study found that the 10-year incidence of cosmetic sequelae following Level I and Level II oncoplastic surgery was 11.5% and 20.0%, respectively (23). In addition, patients with noticeable breast deformities often experience substantial psychological distress, which negatively affects self-esteem and body image, leading to symptoms of anxiety and depression (24). Therefore, achieving optimal aesthetic outcomes not only helps reduce postoperative psychological burden but also significantly improves patients’ overall quality of life (25).

A study by P.Berrino et al. demonstrated that 78% of patients with Grade II deformities who underwent corrective surgery achieved good to excellent cosmetic outcomes (26). Similarly, a retrospective study by Michael S. Chin and colleagues involving 12 patients who received reduction mammoplasty or mastopexy reported satisfactory aesthetic results and acceptable breast symmetry in all cases (27). Sherif Youssif et al. treated 30 patients with post-BCS deformities using flap-based reconstruction methods—including muscle-sparing latissimus dorsi (MSLD) flaps, TDAP flaps, and ICAP flaps—and found a 94% overall satisfaction rate among patients (28). These findings support the conclusion that correcting post-BCS deformities significantly improves breast appearance and, in turn, enhances patients’ quality of life.

In previous studies, a lack of standardized tools for evaluating patient satisfaction and aesthetic outcomes has been noted, with most satisfaction data derived from case series being largely subjective. In response, several objective assessment tools have recently been introduced for evaluating postoperative outcomes. For instance, computer-based programs such as BCCT. Core (Breast Cancer Conservative Treatment: Core) have been widely promoted for the objective scoring of cosmetic outcomes in breast-conserving treatment (29); Digital 3D scanning technologies have also been utilized to quantify morphological changes in breast contour with greater precision (30); Additionally, patient-reported outcome measures (PROMs), such as the BREAST-Q, EORTC QLQ-C30, and QLQ-BRECON23, have been incorporated to assess patient satisfaction and quality of life more comprehensively (31). The use of these tools enables a more accurate and multidimensional assessment of postoperative outcomes from the patient’s perspective.

### 2.4 Factors influencing willingness to undergo deformity correction after breast-conserving surgery

A study conducted by the University of Texas found that among women dissatisfied with their cosmetic outcomes following BCS, 46.2% expressed interest in undergoing breast reconstruction (32). Notably, 70% of participants in the study were of Hispanic descent. Panchal et al. reported on the evolving trends in post-mastectomy breast reconstruction in the United States, noting that by 2015, the average reconstruction rate had risen to 54% (33). Compared to patients in Western countries, who generally demonstrate a stronger desire for correction, Chinese women with post-BCS deformities exhibit relatively low levels of willingness to pursue reconstructive surgery. A multicenter domestic survey reported that only 9.6% of breast cancer patients expressed interest in reconstruction (34). This hesitation may be attributed to a complex interplay of factors, including age, employment status, educational background, household income, financial burden, and awareness or perception of reconstructive options. As shown in **Table 2**.

**Table 2:** Patient-related influencing factors.

Category of factors	Specific factors and manifestations	Tendency	References
Personal and Psychological	Concern for breast appearance	Tends to accept	(36, 60)
	Fear of surgical risks (e.g., complications, anesthesia, recovery time)	Tends to decline	(58, 59)
	Presence of anxiety or depression	Tends to decline	(24, 52)
	High aesthetic expectations	Tends to accept	(60)
Socio-demographic	Age < 50 years	Tends to accept	(34, 36)
	Higher education level	Tends to accept	(40, 43)
	Urban residence	Tends to accept	(38, 39)
	Employed outside the home	Tends to accept	(48)
	Spousal or family support	Tends to accept	(49)
	Religious or cultural concerns	Tends to decline	(43, 44)
Physical and Clinical	Obesity (BMI > 30)	Tends to decline	(46, 47)
	Large tumor size or advanced TNM stage	Tends to decline	(20, 38)
	History of radiotherapy	Tends to decline	(55, 56)
	Moderate to severe deformity (Grade II–III)	Tends to accept	(26, 57)
Healthcare System	Lack of surgical information provided by physicians	Tends to decline or hesitate	(61, 62)
	Lack of insurance coverage for corrective procedures	Tends to decline	(76, 78)

#### 2.4.1 Sociodemographic factors

##### 2.4.1.1 Age

Age is a critical factor influencing the willingness to undergo corrective procedures. According to data from China’s National Cancer Center, the age-standardized incidence of breast cancer peaks in the 45 – 54 age group (35). As the onset of breast cancer shifts toward younger populations, aesthetic expectations after surgery have also increased. One study reported that patients aged 50 years or younger were 4.3 times more likely to undergo reconstruction compared to older individuals (36). In general, older patients tend to prioritize oncologic safety, while younger women are more inclined to pursue aesthetic restoration, making them the primary candidates for deformity correction.

##### 2.4.1.2 Economic status and geographic location

Socioeconomic status and place of residence also significantly affect the likelihood of choosing reconstructive surgery. A study from Western Australia revealed that patients from disadvantaged backgrounds—particularly those with low income or living in rural areas—were less likely to undergo reconstruction (37). Similarly, U.S.-based research showed that patients living in non-metropolitan areas were significantly less likely to have reconstruction compared to their urban counterparts (OR=0.60, *P* < 0.001) (38). Schumacher and colleagues further confirmed that financial constraints play a substantial role in patients’ decision-making (39). Women residing in cities and from families with stronger financial support were more likely to accept corrective surgery.

##### 2.4.1.3 Race and cultural background

One U.S. study found that patients living in communities with lower educational attainment (i.e., where more than 21% of residents had not completed high school) had significantly lower breast reconstruction rates than those in more educated areas (OR=1.152; 95% CI=1.104 – 1.203; *p* < 0.001) (40). Individuals with higher education levels often have a deeper understanding of breast deformity correction and a stronger aesthetic drive. In contrast, those with lower education levels may lack awareness and be more likely to forgo reconstruction. Alderman et al. found that non-White patients—including African American, Hispanic, and Asian women—had significantly lower rates of reconstruction compared to White patients (OR=0.480, *p* < 0.001; OR=0.450, *p* < 0.001; OR=0.290, *p* < 0.001) (41). These disparities likely stem from cultural beliefs, aesthetic norms, and differences in access to healthcare resources. In the decision-making process, Chinese women may be influenced by cultural fatalism and other sociocultural factors, which can lead to resistance toward undergoing surgical procedures (42). Li Zhang conducted a questionnaire-based survey among breast cancer patients in western China and found that only 2.42% of respondents had an in-depth understanding of breast-conserving surgery. The level of awareness was significantly associated with religious belief (*P* < 0.01), with non-religious individuals demonstrating a better understanding of the procedure. This suggests that personal belief systems may, to some extent, influence patients’ acceptance of surgical interventions (43). Ethnic differences were also evident. Angela Li reported that some Hispanic women explicitly expressed, during interviews, their unwillingness to undergo additional surgical procedures, instead insisting on a natural or minimally invasive approach (44). In India, cultural beliefs associated with breast cancer stigma and fear of physical contact often cause patients to experience shame, which hinders their willingness to undergo breast reconstruction or corrective surgery (45).

##### 2.4.1.4 Body mass index

Overweight and obesity—defined as BMI >25 and BMI >30, respectively—are associated with decreased willingness for corrective surgery. This may be due to concerns about postoperative complications, which tend to be more common in obese patients (46). Studies have confirmed a higher rate of surgical complications in this population (47), contributing to their reluctance to undergo secondary procedures.

##### 2.4.1.5 Employment status

Employment status is another relevant factor. One study showed that among patients who did not undergo breast reconstruction, a significantly higher proportion were unemployed (48.9% vs. 21.3%, *P* < 0.001) (48). Employed individuals are generally more concerned with physical appearance, as breast aesthetics can influence self-confidence and social engagement.

##### 2.4.1.6 Spousal support

Spousal support plays a crucial role in reconstruction decision-making. A domestic qualitative study found that a partner’s attitude significantly affected a patient’s willingness to pursue reconstruction (49, 50). P. Ananian’s research revealed that 26% of women living with a partner identified their spouse’s opinion as the most influential factor in decision-making (51). A multicenter study in China also reported that 48.6% of decisions regarding reconstruction were made by spouses. Given the high cost of deformity correction—which is not fully covered by Chinese health insurance—spousal support, both emotional and financial, is essential in shaping a patient’s surgical choice.

##### 2.4.1.7 Psychological factors

A study from Australia found that regret associated with reconstruction decisions was strongly linked to poor body image and psychological distress (52). Research by Toni Zhong further suggested that non-White patients with more pessimistic personality traits were at greater risk of experiencing regret after reconstructive surgery (53).

#### 2.4.2 Disease-related factors

##### 2.4.2.1 Tumor size

Several studies have shown that the likelihood of undergoing breast reconstruction decreases as the size of the invasive tumor increases (41). In China, larger tumors are often perceived by patients as a greater oncologic threat, leading them to prioritize safety over aesthetics. Research by Nava and colleagues (20) also demonstrated that radiation reduces the elasticity of breast tissue and the survival rate of fat grafts, resulting in lower patient expectations for corrective outcomes. Furthermore, large tumor volume may lead to more extensive defects, increased surgical complexity, and greater financial burden, all of which contribute to reduced willingness to undergo correction.

##### 2.4.2.2 TNM stage

Advanced TNM staging is associated with a higher risk of local recurrence and lower disease-free survival, which may influence patients to forgo reconstruction. Studies have confirmed that patients with later-stage disease are less likely to opt for breast reconstruction (38). A U.S. study reported that although women with ductal carcinoma *in situ* (DCIS) or stage I disease represented only 42.1% of the overall cohort, they accounted for 87.6% of those who underwent reconstruction (36). Similarly, a Canadian study found that patients with earlier-stage disease were significantly more likely to receive breast reconstruction.

##### 2.4.2.3 Radiotherapy

Generally, all patients with invasive breast cancer undergoing BCS are recommended to receive whole-breast radiotherapy (54). Waljee et al. found that patients who underwent radiation were more likely to experience postoperative breast asymmetry (55). Radiotherapy can also negatively affect reconstructive outcomes. A study from Michigan revealed that radiation therapy was significantly associated with a higher complication rate (68% vs. 31%, *P* =0.006) and increased reconstruction failure (37% vs. 8%, *P*=0.07) *(* 56). Therefore, concerns about radiation-related complications may reduce a patient’s willingness to undergo corrective procedures.

##### 2.4.2.4 Degree of deformity

Based on Clough’s classification (57), breast deformities after BCS can be divided into three grades: Grade I: The affected breast appears normal but exhibits asymmetry in shape or volume compared to the contralateral side. Grade II: The affected breast has a noticeable deformity that can be corrected through partial reconstruction. Grade III: The affected breast shows severe deformity or diffuse fibrosis, requiring total mastectomy. The severity of deformity often correlates with greater psychological distress, which may either motivate patients to pursue corrective surgery or, conversely, discourage them due to the anticipated difficulty of repair and associated risks.

#### 2.4.3 Other factors

##### 2.4.3.1 Fear and anxiety about surgery

Fear of undergoing a second surgery is one of the most commonly cited reasons for declining corrective procedures (58). Patients may be concerned about surgical risks, potential complications, postoperative pain, or the psychological burden of another operation. Additionally, some patients mistakenly believe that reconstruction could obscure signs of cancer recurrence and hinder disease monitoring. Surgical risks may include anesthesia-related complications, implant rupture, and failure of autologous grafts (e.g., fat necrosis). Concerns over surgical tolerance also play a role in decision-making. A foreign survey on reconstruction preferences showed that among women who wished to undergo reconstruction, 63% feared it might mask signs of recurrence, and 39% worried it might increase the risk of recurrence (59).

##### 2.4.3.2 High expectations for postoperative aesthetic outcome

Many patients hope to restore their preoperative breast appearance through corrective surgery, thereby avoiding the emotional distress caused by wearing prostheses or seeing bodily changes in the mirror. Some seek to reclaim a sense of normalcy and femininity. In a multicenter qualitative study comparing mastectomy and oncoplastic BCS, one of the most influential factors in patient choice was the desire to preserve or restore feminine identity and appearance postoperatively (60).

##### 2.4.3.3 Surgeons’ attitudes toward deformity correction

Surgeons’ attitudes toward reconstruction can significantly affect patients’ willingness to undergo deformity correction. A nationwide survey in Japan found that 31.3% of breast surgeons never provided patients with information about reconstructive options (61). Similarly, Alderman et al. highlighted the critical role of physicians in providing comprehensive information (62). Surgeons may selectively inform patients based on personal biases, such as age or economic status. A study in Saudi Arabia showed that 76.5% of surgeons did not recommend breast reconstruction out of concern that it might obscure signs of local recurrence (63). These findings suggest that some surgeons’ limited understanding of reconstructive techniques may inadvertently discourage patients from considering correction.

### 2.5 Types of corrective procedures for postoperative breast deformity

For patients with a strong desire for correction, selecting an appropriate surgical approach is crucial. Common techniques include: 1. Flap-based reconstruction – such as local tissue advancement flaps, latissimus dorsi (LD) flaps, thoracodorsal artery perforator (TDAP) flaps, and deep inferior epigastric perforator (DIEP) flaps; 2. Reduction mammaplasty, mastopexy, or implant placement; 3. Autologous fat grafting or liposuction; 4. Scar revision or secondary reconstruction modifications. As shown in **Table 3**. Selection should consider various factors, including defect size and location, breast volume, and surgeon expertise. Studies indicate that for patients with unsatisfactory cosmetic results, local flap reconstruction and fat grafting are more commonly used on the affected side, while reduction mammaplasty is more often performed on the contralateral breast to improve symmetry (64). Autologous fat grafting is especially promising due to its minimal scarring and relatively low complication rates (65). It is generally preferred for small-volume defects, whereas flap reconstruction or reduction/mastopexy with contralateral adjustment is recommended for larger defects. Currently, no universally accepted standard exists for selecting the optimal surgical approach, and evidence-based guidelines are yet to be established.

**Table 3:** Comparative overview of deformity correction techniques.

Procedure	Advantages	Disadvantages	Indications
Local Flap Transfer (e.g., LD flap)	Good blood supply, can fill larger defects	Significant surgical trauma, potential large scarring and functional	Moderate to large breast tissue defects; unsuitable for implant or fat reconstruction; requires relatively sufficient local tissue
Distal Flap (e.g., abdominal muscle flap)	Sufficient volume, can correct large deformities simultaneously	Longer procedure, higher risk of long-term complications	Large tissue defects requiring substantial volume reconstruction
Autologous Fat Grafting	Maintains natural feel; minimally invasive, no visible incisions, natural shape	Varying fat survival rates; limited volume of grafts; potential for fat necrosis or complications post-op	Small volume deficiencies, poor breast contour; patients with moderate BMI
Contralateral Breast Correction	Improves symmetry between both breasts, enhances overall aesthetics	Increases surgical scope, may require additional recovery time	Significant asymmetry in contralateral breast after breast-conserving surgery
Breast Reduction/Fixation	Improves ptosis, restores breast contour and symmetry, suitable for larger breasts	Reduces glandular tissue, potential for nipple sensation loss or scarring	Moderate to large breasts with ptosis, mild to moderate volume asymmetry or deformity
Biological Fillers	Simple procedure with no donor site damage	High cost, risk of foreign body infection	Thin patients or those with insufficient local tissue; seeking minimally invasive options

LD flap, Latissimus Dorsi flap.

The extent of the defect is categorized based on the proportion of total breast volume lost. A loss of less than 15% is considered a small defect, 15% to 30% is considered a moderate defect, and greater than 30% is considered a large defect. This can also be assessed in conjunction with the extent of anatomical structure damage.

In addition to surgical repair, a comprehensive patient-centered strategy should incorporate psychological support. As shown in **Figure 1**. Surgical treatment can alter body image, which in turn influences both the patient’s willingness to undergo correction and the choice of procedure. Surgeons should explore patients’ perspectives on appearance and body image (66), and consider preoperative cognitive behavioral therapy or stress management interventions (67), Postoperative body image education and peer support groups can further facilitate recovery. Artificial intelligence (AI) and deep learning also show promise in preoperative planning and outcome prediction (68). For example, AI-based breast imaging analysis can simulate postoperative contours and guide personalized surgical planning (69). Three-dimensional surface imaging (3D-SI) provides objective quantification of cosmetic outcomes (30). Such tools may significantly enhance the precision and personalization of deformity correction.

**Figure 1:**
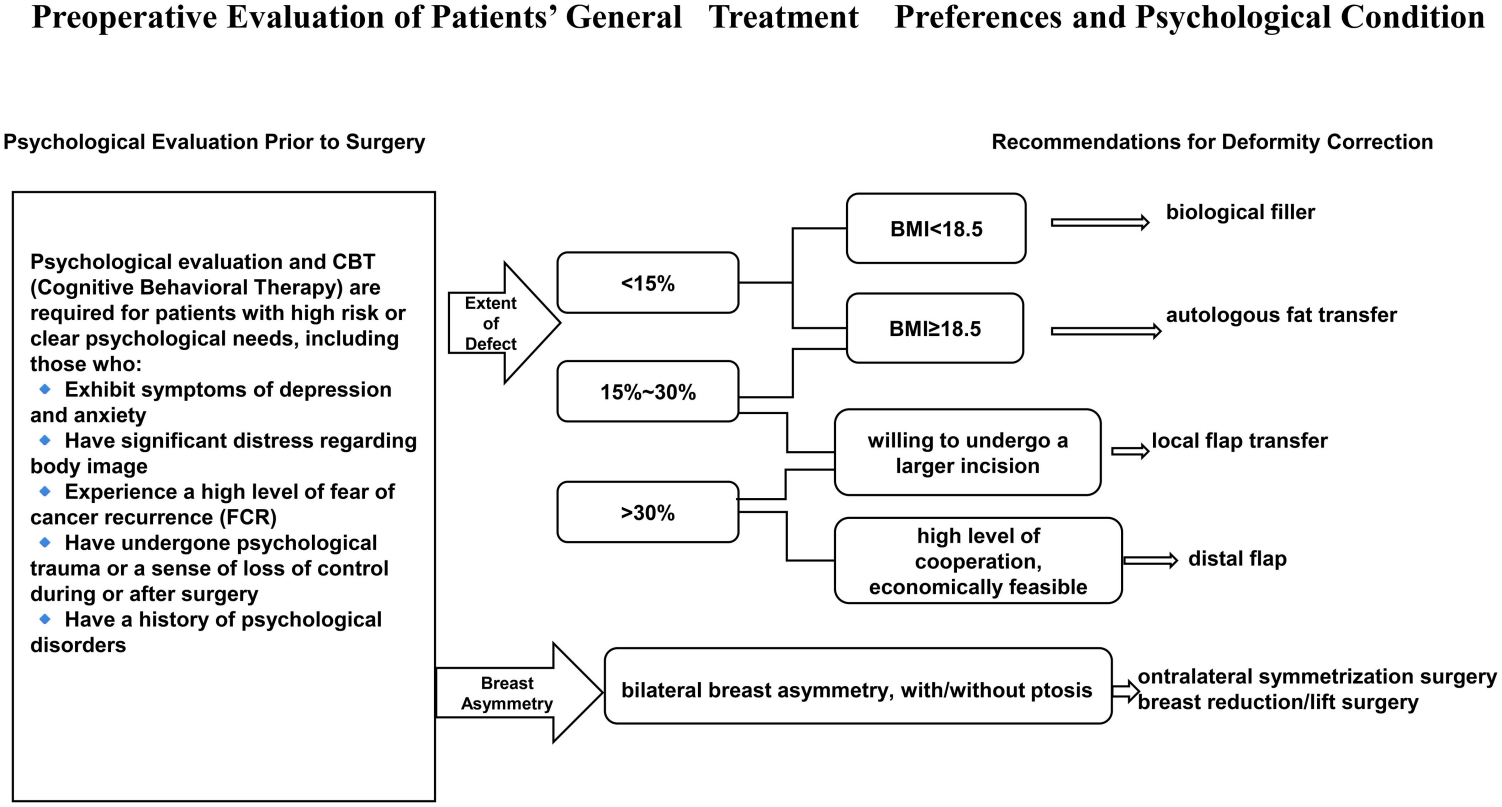
Preoperative evaluation of patients’ general treatment preferences and psychological condition.

### 2.6 Clinical practice recommendations and strategies

To address the multifaceted factors influencing Chinese women’s willingness to undergo corrective surgery after BCS, several practical strategies should be implemented—ranging from physician communication and public education to policy reform and insurance coverage.

First, medical professionals should enhance health education regarding breast deformity correction and diversify the channels through which patients receive information. Lack of knowledge or misconceptions may deter patients from pursuing corrective procedures. Surgeons should use verbal explanations, visual aids, internet resources, and videos to communicate accurate, evidence-based information (70). In recent years, shared decision-making (SDM) and patient education have been increasingly emphasized (71). When multiple surgical options are available, patients should be provided with counseling services and actively involved in treatment planning. Surgeons must also take patients’ aesthetic preferences into account—for example, offering tailored reconstructive approaches based on different breast shapes—and consider simultaneous contralateral surgery (e.g., reduction or mastopexy) to achieve symmetry.

Second, surgeons should implement personalized psychological interventions. Existing studies show that cognitive behavioral therapy (CBT) aimed at reducing body image anxiety can significantly improve postoperative adjustment (67). Fear of cancer recurrence (FCR) is a major concern among survivors and is defined as “fear, worry, or concern about cancer returning or progressing.” (72). Simard et al. found that FCR negatively impacts emotional well-being, quality of life, and daily functioning. Tailored CBT interventions have been shown to significantly reduce FCR and related maladaptive behaviors (73). These interventions have demonstrated clinical efficacy and feasibility in large randomized controlled trials, such as the SWORD study (74), and case reports have further illustrated their practical application (75). Introducing such interventions in China would represent both a clinical advancement and a systemic challenge.

Finally, more attention should be paid to spousal support and healthcare policy. Partners should be encouraged to respect and support patients’ decisions, empowering them to pursue surgery with confidence. Economic concerns are among the primary reasons for declining reconstruction (76), underscoring the importance of financial support from spouses and families. The Women’s Health and Cancer Rights Act (WHCRA), enacted in the United States in 1998, significantly strengthened the protection of patients’ rights to undergo breast reconstruction. A study by Rachel L. Yang et al. demonstrated that following the implementation of this legislation, the rate of immediate breast reconstruction increased by 4.2-fold among Medicaid patients, 2.9-fold among Medicare patients, 2.6-fold among privately insured patients, and 2.1-fold among self-paying patients (77). A study from Fudan University Shanghai Cancer Center indicated that the low rate of breast reconstruction in China may be related to limited medical resources. At present, most cancer hospitals in China do not have plastic surgery departments, and the number of breast surgeons is relatively low with heavy workloads, which hinders the application of reconstructive techniques and prolongs the learning curve (78). Based on this, National health authorities should consider expanding insurance coverage for deformity correction procedures and improving resource allocation to help alleviate financial barriers and improve access to care.

## 3 Conclusion and outlook

With the widespread adoption of breast-conserving surgery (BCS), increasing attention has been paid to postoperative breast aesthetics and quality of life. Although BCS has been widely validated for its oncologic safety, the incidence of postoperative breast deformities remains non-negligible, with significant implications for patients’ psychological health and social functioning. Current evidence supports that deformity correction procedures are safe and effective, offering substantial improvements in breast appearance, self-image, and quality of life. However, the overall correction rate in China remains low due to various barriers, including patient-specific factors, unequal access to healthcare resources, and insufficient postoperative education.

Most current research in this field relies on retrospective case series, lacking large-scale, multicenter, prospective randomized trials (17). Follow-up durations and evaluation methods are inconsistent, and no universally accepted aesthetic assessment standard exists (29). Furthermore, standardization of surgical training and multidisciplinary collaboration remain underdeveloped in China, and psychosocial interventions for postoperative patients are underrepresented in the literature (79). Moving forward, greater emphasis should be placed on multidisciplinary collaboration and the integration of breast surgery with reconstructive and psychological care. Establishing individualized assessment systems, improving clinical education and patient counseling, and reforming insurance policies are all essential steps. This article specifically focuses on the perspectives and considerations of Chinese women, which limits the generalizability of certain factors. Further evaluation and systematic investigation are still needed. In addition, high-quality prospective studies are urgently needed to evaluate the long-term oncologic and cosmetic outcomes of different correction techniques, laying the foundation for standardized clinical pathways and evidence-based guidelines.
